# Sertraline-Induced Optic Nerve Dysfunction

**DOI:** 10.7759/cureus.36976

**Published:** 2023-03-31

**Authors:** Ismail Abuallut, Ahmad Y Alqassim, Rubuah Ayyashi

**Affiliations:** 1 Department of Ophthalmology, Faculty of Medicine, Jazan University, Jazan, SAU; 2 Department of Family and Community Medicine, Faculty of Medicine, Jazan University, Jazan, SAU; 3 Department of Ophthalmology, Abha Maternity and Children Hospital, Abha, SAU

**Keywords:** side effects, fluoxetine, sertraline, retinopathy, papilledema, optic neuropathy

## Abstract

This case report describes a rare case of Sertraline-induced optic nerve dysfunction with optic disc edema (papilledema) in both eyes in a 32-year-old male who was on chronic sertraline therapy for the treatment of generalized anxiety disorder and three panic episodes. The patient was presented to our ophthalmology clinic with two bubbles with dark borders in both eyes on the far side for a few months. An optical coherence tomography showed that retinal nerve fiber layer thickness was 98 microns in OD (right eye) and 105 microns in OS (left eye). Another optical coherence tomography findings in both eyes were the elevation of superior and inferior quadrants. Optical coherence tomography findings supported the diagnosis of optic disc edema (papilledema) in both eyes. Magnetic resonance imaging of the brain revealed symmetrical enlargement in the optic nerves (8 mm in diameter at its thickest point). However, abnormal enhancement was absent, excluding optic neuritis. Sertraline was discontinued and replaced by fluoxetine 20 mg. Five months later, papilledema was resolved. On follow-up one month later, the patient continued to improve in terms of symptoms and test results. The case presented demonstrates a rare association between sertraline use and optic nerve dysfunction. Adding to the increasing number of patients using sertraline worldwide, further research is warranted to investigate the incidence of this association and explore possible pathologic mechanisms.

## Introduction

Selective serotonin reuptake inhibitors (SSRIs) are commonly used to treat a variety of mental disorders, including major depressive disorder and generalized anxiety disorder [1]. Generalized anxiety disorder (GAD) is a chronic mental illness characterized by excessive, uncontrollable, constant worry and stress. Common somatic presentations of GAD may include palpitation, tremor, restlessness, and nausea, among others. Symptoms should cause a significant functional deficiency in various areas of productivity, family life, and socialization, impacting the individual's quality of life [2,3]. Sertraline is one of the most widely used SSRIs, with over 11 million prescriptions written in the United Kingdom alone in 2016 [4]. Sertraline may cause side effects, such as stomach problems, nausea, weakness, and headache [5]. Despite its regular use, sertraline has been linked to a small number of ocular side effects, including optic neuropathy and acute angle-closure glaucoma [6,7]. Also, there are five reported cases of suspected sertraline-associated maculopathy [8-12]. A case series of three patients from different age groups ranging from 27 to 68 years showed that patients on sertraline suffered from diminished visual acuity. In the same case series, all patients developed maculopathy following sertraline use [13]. In the current report, we describe a rare case of sertraline-induced optic nerve dysfunction.

## Case presentation

A 32-year-old male presented to our ophthalmology clinic with two bubbles with dark borders in both eyes on the far side for a few months. The patient noticed visual field defects and floaters. There was no loss of vision, pain, redness, double vision, tearing, dryness, foreign body sensation, discharge, eyelid crusting, eyelid swelling, ptosis, flashes, or halos. The patient had no fever, night sweats, fatigue, or unintended changes in weight and appetite. The patient also was not aware of any previous similar conditions, nor he gave a history of previous hospital admission, blood transfusion, or similar condition in the family. The patient reported that he had a history of high myopia (-11 diopter in both eyes), unspecified papilledema, optic nerve edema, and temporal arthritis involving the left eye and right eye. He also mentioned corrective lens use and laser therapy. He denied recent eye trauma or infection. Appendectomy and white coat hypertension was discovered through a review of other systems. Before the onset of symptoms, the patient had been taking sertraline (Zoloft) 25 mg tablet orally four times per day (QID) for 32 months for the treatment of generalized anxiety disorder and three panic episodes. The patient was on Ketotifen (Zaditor) 0.025% (0.035) eye drops.

Upon clinic arrival, the patient was conscious, alert, and oriented to time, place, and person. On examination, both eyelids were in normal position and margin. Conjunctiva was white and quiet in both eyes. Snellen chart test for visual acuity was 20/25 OD (oculus dexter, i.e., right eye) and 20/20 OS (oculus sinister, i.e., left eye). Visual field test results demonstrated full confrontation in both eyes. His intraocular pressure measured 15 mmHg bilaterally, which is within the normal range. Pupils were regular, rounded, and showed normal size. Slit-lamp examination revealed a clear cornea, clear lens, deep and quiet anterior chamber, and normal iris without rubeosis in both eyes. An ophthalmoscopic examination of the optic disc revealed disc edema in both eyes. An ophthalmoscopic examination of the retina and vessels of both eyes revealed clear vitreous without hemorrhage, cells or pigment, normal vessels, normal macula contour without heme, edema, drusen or exudate, and normal appearance of periphery without retinal tears, breaks, holes, or mass.

The laboratory examination showed a low mean corpuscular volume (MCV) of 76.1 femtolitres (fL), low mean corpuscular hemoglobin (MCH) of 25.1 picograms (pg), high glucose of 102 milligrams per deciliter (mg/dL), high blood urea nitrogen (BUN) of 23 mg/dl, high BUN/Creatinine ratio (26 Ratio), high carbon dioxide (CO2) of 33 milliequivalents per liter (mEq/L), high total protein (8.5 gram/dL), high Albumin (5.1 gram/dL), high ANION GAP (20 mEq/L), high sedimentation rate by modified Westergren of 30 millimeters per hour (mm/h), high red blood cells (RBC) of 6.06 million per cubic millimeter (mil/uL), high platelet count of 366 kilos per microliter (K/UL), high C-reactive protein (17.5 mg/L), low potassium (3.1 mEq/L), and low chloride (87 mEq/L). All other tests were negative, including antinuclear antibodies (ANA screen IFA W/REFL titer and pattern) test, Lyme disease antibodies immunoglobulins G and M (IgG and IgM) immunoblot blood test, and human immunodeficiency virus (HIV) 1/2 antigen/antibody, fourth generation W/RFL test.

**Figure 1:**
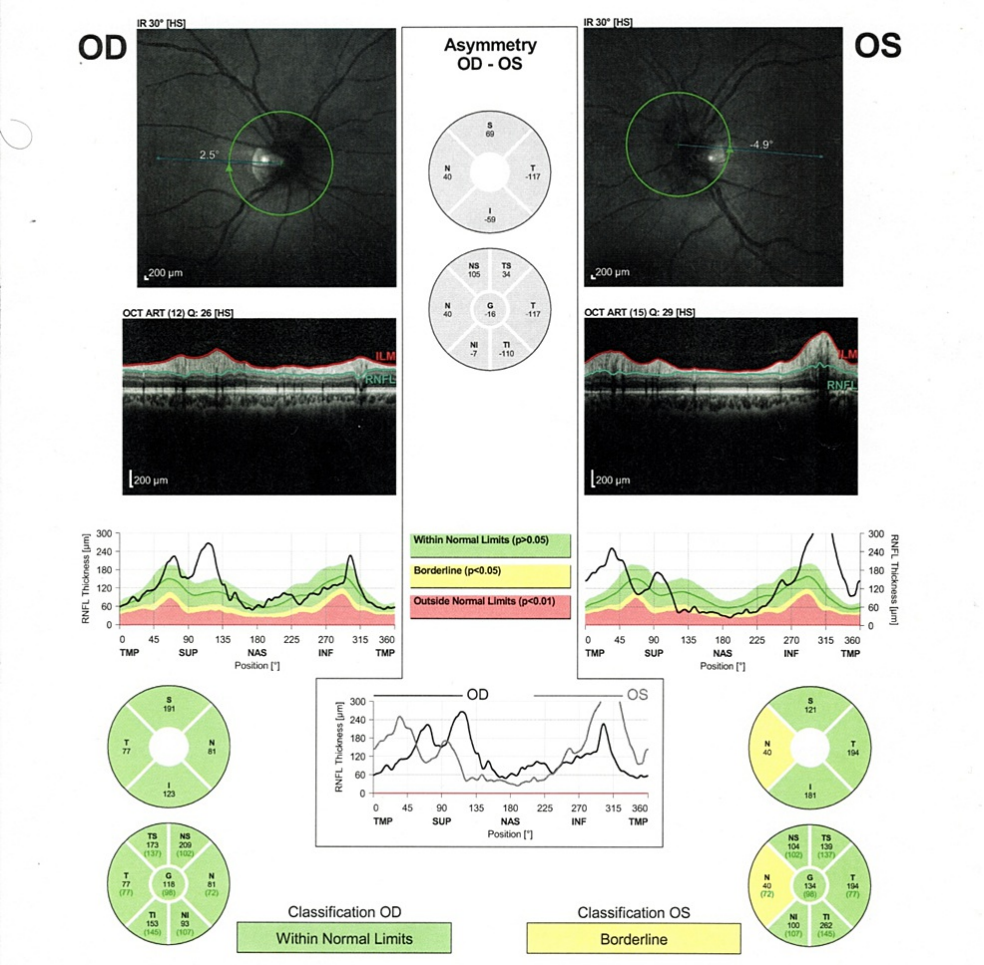
An optical coherence tomography (OCT) shows that retinal nerve fiber layer thickness was 98 microns in OD and 105 microns in OS. Other findings in both eyes were the elevation of superior and inferior quadrants. Those findings supported the diagnosis of optic disc edema (papilledema) in both eyes.

**Figure 2:**
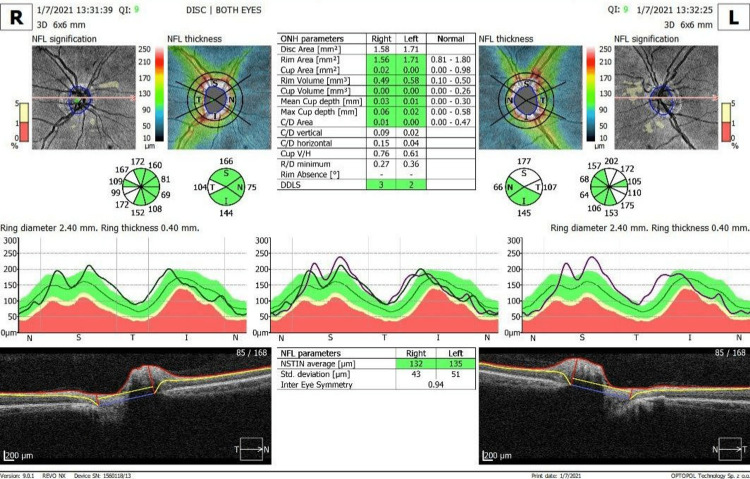
At five months follow-up after discontinuation of sertraline, the papilledema resolved.

An optical coherence tomography (OCT) showed that retinal nerve fiber layer thickness was 98 microns in OD and 105 microns in OS (Figure 1). Another OCT findings in both eyes were the elevation of superior and inferior quadrants. OCT findings supported the diagnosis of optic disc edema (papilledema) in both eyes. MRI Brain revealed symmetrical enlargement in the optic nerves (8 mm in diameter at its thickest point). However, abnormal enhancement was absent, excluding optic neuritis. Sertraline was discontinued and replaced by fluoxetine 20 mg. Five months later, papilledema resolved (Figure 2). On follow-up one month later, the patient continued to improve in terms of symptoms and test results.

## Discussion

In this case, we highlight a rare case of sertraline-induced optic nerve dysfunction, adding to the nine published cases in the literature. In our case, the patient presented with optic nerve edema, which is consistent with what has been found by other authors [6,7,10,14-16]. The majority of previous studies reported intraocular pressure changes [14], however, the patient’s intraocular pressure was normal in our study. There was an unexpected connection between sertraline use and a possible sertraline-related maculopathy in one case report [8] and one case series [13], which was not found in this case. Contrary to our findings, two previous authors found a relationship between sertraline and central scotoma [7,10]. Inflammatory markers are usually absent in such cases but were high in our case. OCT findings in our case demonstrated a retinal nerve fiber layer thickness of 98 microns in OD and 105 microns in OS, confirming the diagnosis of papilledema, which is in agreement with two other case reports [9,13]. In the current case, MR Brain WWO revealed symmetrical enlargement in optic nerves but did not demonstrate abnormal enhancement, which excluded optic neuritis. The case was managed by sertraline cessation which improved the patient’s symptoms. This is consistent with what has been found in the case series [13]. The finding of improved visual acuity after discontinuing sertraline in this case is consistent with some case reports [13] but not with others [8,13,17]. Sertraline-related maculopathy is thought to be caused by several mechanisms. According to one study [9], improved serotonin bioavailability enhances phospholipase C activation through the 5HT2A receptor [18]. This could increase the intracellular reactive oxygen species activity and, eventually, retinal degeneration. Increased amounts of serotonin in the central and peripheral nervous systems can interact negatively with RPE and photoreceptor serotonin receptors through a secondary messenger mechanism involving cyclic adenosine monophosphate (cAMP) [12,19]. This could lead to ganglion cell death and the formation of maculopathy [20]. Until a fully understood mechanism is identified, further research on the serotonin receptor profile and function of the RPE is warranted. Physicians should be aware of this uncommon but potentially reversible ocular complication of sertraline use. We advise patients taking sertraline to have a regular ophthalmologic check-up.

## Conclusions

The case presented demonstrates a rare association between sertraline use and optic nerve dysfunction. Given the increasing number of patients using sertraline worldwide, further research is warranted to investigate the incidence of this association and explore possible pathologic mechanisms.
